# Stability of Bioactive Compounds in Olive-Pomace Oil at Frying Temperature and Incorporation into Fried Foods

**DOI:** 10.3390/foods10122906

**Published:** 2021-11-24

**Authors:** María-Victoria Ruiz-Méndez, Gloria Márquez-Ruiz, Francisca Holgado, Joaquín Velasco

**Affiliations:** 1 Instituto de la Grasa (IG), Consejo Superior de Investigaciones Científicas (CSIC), Campus/Bd 46, Ctra. de Utrera km 1, 41013 Sevilla, Spain; jvelasco@ig.csic.es; 2 Instituto de Ciencia y Tecnología de los Alimentos y Nutrición (ICTAN), Consejo Superior de Investigaciones Científicas (CSIC), José Antonio Novais, 10, 28040 Madrid, Spain; gmarquez@ictan.csic.es (G.M.-R.); f.holgado@ictan.csic.es (F.H.)

**Keywords:** frying, French fries, nuggets, croquettes, olive-pomace oil, triterpenic alcohols, oleanolic acid, squalene, sterols, tocopherols, aliphatic alcohols

## Abstract

The stability of minor bioactive compounds in olive-pomace oils (OPOs) was evaluated at frying temperature under the conditions of a thermoxidation test. Bioactive compounds analyzed included squalene, tocopherols, sterols, triterpenic acids and alcohols, and aliphatic alcohols. In order to determine the amount of OPO bioactive compounds incorporated into foods after frying, three different kinds of frozen products were selected, i.e., pre-fried potatoes (French fries), pre-fried battered chicken nuggets, and chicken croquettes (breaded patties), and were used in discontinuous frying experiments. Results obtained in both the thermoxidation and frying studies showed high stability of triterpenic alcohols (erythrodiol and uvaol), oleanolic acid, and aliphatic alcohols, naturally present in OPOs. In all fried foods, the content of lipids increased after frying, as expected, although the extent of absorption of OPOs into fried foods and the exchange with food lipids depended on the food characteristics. Overall, frying with OPOs improved the nutritional properties of all products tested by increasing the level of oleic acid and by the incorporation of squalene, triterpenic acids and alcohols, and aliphatic alcohols, in significant quantities.

## 1. Introduction

Olive-pomace oil (OPO) is the main by-product of virgin olive oil production. It is obtained mainly by solvent extraction from the olive cake and refined before consumption. All olive-extracted oils, i.e., virgin olive oils, refined olive oils, and olive-pomace oils, the latter two commercialized after blending with variable amounts of virgin olive oil, are essential components of the Mediterranean diet, to which fried foods contribute greatly. Results of the comparative behavior of olive-extracted oils and other vegetable oils reflect that all olive-extracted oils show great stability to thermal oxidation and are highly suitable for frying, due to their fatty acid composition, being rich in monounsaturated and poor in polyunsaturated fatty acids, and occurrence of minor compounds with protective effects. Among olive-extracted oils, frying performance of OPOs has been much less studied than that of virgin and refined olive oils even though it is, likewise, a good alternative for frying [1]. In the case of virgin olive oils, their remarkable resistance to frying temperatures is attributed to their composition, being rich in monounsaturated and poor in polyunsaturated fatty acids, and the presence of antioxidants such as α-tocopherol, phenolic compounds, squalene, and Δ5-avenasterol as anti-polymerization agent [2,3]. However, when virgin olive oils were used for repeated frying operations, their phenolic contents and, therefore, their main antioxidant protection was found to decrease significantly [4,5], while sterols, squalene and triterpenic alcohols (erythrodiol and uvaol), and acids (oleanolic and maslinic acids) showed high stability at frying temperature [6]. 

The scarce studies published so far on the changes of OPOs during heating or frying focused on the modifications produced in the glyceridic fraction, primarily triacylglycerols, and the products formed [7,8,9]. However, to our knowledge, no reports have been published on how frying conditions affect the naturally occurring bioactive compounds present in the minor, unsaponifiable fraction of OPOs. As a derivative of the olive fruit, OPOs are characterized by a fatty acid composition rich in monounsaturated fatty acids, specifically oleic acid, and elevated contents of bioactive compounds, presenting the highest amounts of phytosterols, triterpenic compounds, and aliphatic fatty alcohols found in all categories of olive oils [10]. The content of total sterols in OPOs is always higher than 2500 mg/kg and aliphatic alcohols exceed 1500 mg/kg [1]. A wide range and higher content than 120 mg/kg can be found in triterpenic acids, mainly composed of oleanolic acid, and up to 500 mg/kg in triterpenic alcohols, depending on the refining conditions [11]. In addition, relevant quantities of squalene and tocopherols are found in OPOs [1,12]. All these minor compounds are of utmost importance from a nutritional point of view, since they can induce positive effects on inflammatory pathological processes and cardiovascular health [13].

Studies focused on the health effects of those compounds most characteristic of OPOs, i.e., triterpenic alcohols and aliphatic fatty alcohols, have shown that the former, i.e., erythrodiol and uvaol, seem to protect from cardiac hypertrophy [14] and to exert antiatherogenic [15], anti-inflammatory [16], anti-hypertensive [17], and neuroprotective effects [18], while aliphatic fatty alcohols’ intake improved the lipoprotein profile in humans [19] and showed anti-inflammatory activity [20].

With regard to the incorporation of oil bioactive compounds to fried foods, different oils have been used in the studies published so far, as reviewed by Chiou and coworkers [3,21]. They showed and discussed the data obtained regarding the retention of tocopherols, phytosterols, polyphenols, squalene, and carotenoids in French fries prepared with a variety of vegetable oils, including virgin and refined olive oils [21]. The authors later highlighted the potential of virgin olive oils for improving health properties of fried foods through incorporation of phenolics, tocopherols, phytosterols, squalene, and triterpenic acids [3]. Recently, Alzaa et al. remarked on the positive effects of using extra virgin olive oil vs. canola and grapeseed oils for frying potato chips, chicken nuggets, and broccoli, based on the foods’ enrichment with antioxidants and monounsaturated fatty acids [22]. However, to our knowledge, no study has been carried out with OPO in this context so far. 

In this work, the stability of bioactive compounds of OPOs was evaluated at frying temperature using a thermoxidation test. Moreover, three types of frozen products of vegetal and animal origin, including coating and/or pre-fried treatments, were selected to determine the amount of OPO bioactive compounds retained after frying. Thus, pre-fried stick potatoes (French fries), pre-fried, battered chicken nuggets, and chicken croquettes (breaded patties) were fried under discontinuous conditions, thus simulating the frying procedure used in households, restaurants, and frying outlets. Analyses of the oils and lipids extracted from the fried foods included characterization and quality determinations and quantification of squalene, sterols, tocopherols, triterpenic acids and alcohols, and aliphatic alcohols.

## 2. Materials and Methods

### 2.1. Chemicals

The 5-α-Cholestan-3-ol, heneicosanol, betulinic acid, squalane, and tocopherols (α, β, γ, δ) standards (Purity 99%) were supplied by Sigma-Aldrich SA (St. Louis, MO, USA). *N*,*O*-Bis (trimethylsilyl) trifluoroacetamide (BSTFA) containing 1% trimethylchlorosilane (TMCS) was purchased from Supelco (Bellefonte, PA, USA) and used as a silylation reagent. All other chemicals and reagents were of analytical grade and were acquired from local suppliers. 

### 2.2. Samples

Olive-pomace oils (OPO1, OPO2, and OPO3) were obtained from local suppliers. Frozen pre-fried stick potatoes (French fries), frozen pre-fried, battered chicken nuggets, and frozen chicken croquettes were bought in local markets.

### 2.3. Assays

#### 2.3.1. Thermoxidation

The thermoxidation assay was carried out following the procedure developed by Barrera-Arellano et al. [23]. Three tubes were used for each oil, with 8 ± 0.01 g of oil each. The tubes were heated at 175 °C for 20 h and samples were taken at 2, 5, 8, 10, 15, and 20 h. To simulate discontinuous frying, the tubes remained open and no air flow was applied. The samples were collected in triplicate and stored under nitrogen atmosphere at −20 °C until analysis.

#### 2.3.2. Discontinuous Frying 

Three domestic deep fryers (Moulinex Easy Pro, Model F59-M) of 3-L oil capacity were used to fry four batches of 100 g of frozen foods. The oil surface area-to-volume ratio in the fryers was 0.13 cm^−^^1^. The temperature was controlled by a K-type thermocouple coupled to a recorder, so that each frying started at 175 ± 3 °C. The initial heating period of the oil was set at 5 min. After each frying operation, the basket was shaken and held draining for 1 min to remove excess oil. 

Foods were fried for 10 min and intervals of 2 h were established between frying operations. Experiments were performed in triplicate. No fresh oil was added during the experiments. Samples of frying oil and fried food samples were kept under nitrogen atmosphere and stored at −18 °C until analysis.

### 2.4. Analytical Procedures

#### 2.4.1. Oil Analyses

The methods outlined in the EEC Regulation [10] were used to analyze the fatty acid composition, as well as the sterols’ and triterpenic and aliphatic alcohols’ contents and composition.

Fatty acid composition was determined after transesterification of the oils with KOH in methanol. Fatty acid methyl esters (FAME) were analyzed using a Hewlett-Packard 6890 Series gas chromatograph (GC) equipped with a DB-23-fused silica capillary column (60 m × 0.25 mm, i.d. 0.25-μm film thickness) and a flame ionization detector (FID). The detector and injector temperatures were held at 240 °C while the column temperature remained isothermal at oven temperature of 185 °C. The FAME were calculated based on the retention times and peak areas with a rapeseed oil reference mix. Each fatty acid was expressed as a percentage of mass fractions.

The analysis of the total content and composition of sterols and triterpenic alcohols and aliphatic alcohols were performed in the unsaponifiable fraction of 5 g of oil, as indicated in the EEC Regulation [10] by using 5-α−cholestan-3-ol and n-heneicosanol as internal standards, respectively. After drying, the unsaponifiable matter was separated by thin layer chromatography (TLC) using a mixture of hexane: diethyl ether (87:13, *v*/*v*). After separation, the bands corresponding to aliphatic, sterols, and triterpenic alcohols were scratched off and extracted with chloroform and diethyl ether. The solutions were evaporated to dryness, derivatized with 500 μL of a 1:3:9 (*v*/*v*/*v*) trimethylchlorosilane: hexamethyldisilazane: pyridine solution and analyzed by GC. Once isolated, the different fractions were analyzed in an AT 7890A chromatograph (US10826031), equipped with a split/splitless injector and a FID detector. In all of the cases, a 0.25-mm (i.d.) × 30-m bonded-phase 5% diphenyl-95% dimethylpolysiloxane column with a 0.25-mm film thickness (HP-5MS column, Agilent, prod. No. 19091 J-413) was used. Hydrogen was used as the carrier gas at a constant pressure of 15 psi. The different GC parameters and chromatograms of the different fractions obtained are shown as Supplementary Material (Figures S1 and S2).

Squalene was analyzed following a proven method based on the IOC Standard Method for Determination of the content of waxes, fatty acid methyl esters, and fatty acid ethyl esters by capillary gas chromatography using 3 grams of silica [24]. Briefly, 100 mg of oil added with squalane as internal standard was fractionated by silica column chromatography using a mixture of hexane and diethyl ether 99:1 (*v*/*v*) and then analyzed by GC using a 5% diphenyl-95%dimethylpolysiloxane HP5 column (15 m × 0.25 mm, i.d. 0.25-μm film thickness) and a FID. Hydrogen was used as the carrier gas at a constant pressure of 15 psi. The detector temperature was 350 °C while column temperature program was 80 °C for 1 min, 20 °C/min up to 240 °C, and 5 °C/min up to 350 °C. 

Triterpenic acids were determined following the method proposed by Pérez-Camino and Cert [25], which consists of the isolation of the fatty acid fraction by SPE-NH_2_ columns, silylation of the extracts, and quantitation by gas chromatography (Mod. 7890A, Agilent Technologies), using betulinic acid as internal standard using a 5% diphenyl-95% dimethylpolysiloxane HP5 column (30 m × 0.25 mm, i.d. 0.25-μm film thickness) and a FID. Hydrogen was used as the carrier gas at a constant pressure of 15 psi. The detector temperature was 350 °C while column temperature was held at 260 °C for 30 min. 

Tocopherols were determined by HPLC with fluorescence detection following IUPAC Standard Method 2.432 [26]. The oil samples were dissolved in n-heptane at a concentration of 50 mg·mL^−1^ and analyzed in an Agilent 1260 Infinity HPLC chromatograph (Agilent Technologies, Santa Clara, CA, USA). The chromatograph was equipped with a quaternary pump VL (G1311C), a standard autosampler (G1329B), a thermostatted column compartment (TCC) (G1316A), and a fluorescence detector (FL) (G1321A). A silica HPLC column (LiChrospher^®^ Si 60, 250 mm × 4 mm i.d., 5-µm particle size) (Merck, Darmstadt, Germany) was used. The volume of the sample analyzed was 20 µL. The temperature of the TCC was set at 25 °C. The separation of tocopherols was performed using n-heptane:isopropanol (99:1, *v*/*v*) with a flow rate of 1 mL·min^−1^. The excitation and emission wavelengths in the detector were 290 nm and 330 nm, respectively. Quantification was made by external calibration using tocopherol standards.

The amount of polar compounds was determined by silica column chromatography following IUPAC method 2.507 [26]. Briefly, starting from 1 g of oil, a nonpolar fraction containing unoxidized triacylglycerols was first eluted with a mixture of hexane and diethyl ether 90:10 (*v*/*v*) and then a second fraction, comprised of total polar compounds was eluted with diethyl ether. The polar fraction was further analyzed by high-performance, size-exclusion chromatography, as previously described [27], and oxidized triacylglycerol monomers, triacylglycerol dimers, triacylglycerol oligomers, diacylglycerols, and free fatty acids were determined. Polar fractions were dissolved in tetrahydrofuran at 20 mg·mL^−1^ and analyzed in an Agilent 1200 Infinity Series liquid chromatograph equipped with a quaternary pump VL (G1311C), a standard autosampler (G1329B), a thermostatted column compartment (TCC) (G1316A), and a refractive index detector (G1362A). The separation was performed on two 100- and 500-Å columns (25 cm × 0.77 cm i.d.) packed with porous, highly cross-linked styrene-divinylbenzene copolymers (Agilent Technologies, Palo Alto, CA, USA) connected in series, with tetrahydrofuran (1 mL·min^−1^) as the mobile phase.

Polymers (triacylglycerol dimers and higher oligomers) were analyzed directly by high-performance, size-exclusion chromatography following IUPAC method 2.508 [26] under the same chromatographic conditions described above.

#### 2.4.2. Food Analyses

(a) Moisture content
  Moisture content of initial and fried foods was determined gravimetrically by drying in an oven according to Official AOAC method 7.003 [28].
(b) Lipid content
  Initial and fried foods were frozen, freeze-dried, and ground. The total lipids were obtained by Söxhlet extraction with hexane for 6 hours, according to method UNE 55-062-80 [29].
(c) Food lipid analyses
  The lipids extracted from the initial and fried foods were analyzed following the same methods described in 2.4.1.

### 2.5. Statistical Analyses

The frying and thermoxidation tests were performed in triplicate. The results showed represent the arithmetic mean followed by the standard deviation of three analytical determinations performed, respectively, on independent samples. For the comparison of two means, Student’s t-test was applied, while for three or more, one-factor analysis of variance (ANOVA) and Tukey’s test were used to establish differences between means. A significance level of 5% was considered. Statistical analysis was performed with the SPSS 27 statistical program (IBM).

## 3. Results and Discussion

### 3.1. Characterization and Quality of Unused Oils

Table 1 shows characterization parameters of the OPOs used. 

All oils were fresh and of good quality, with acidity values equal to or less than 0.1% (data not shown) and low contents of oxidized triacylglycerols, which ranged between 1.2% and 1.3%. However, the total contents in the polar compounds were higher than 7.5% in all oils due to the considerable contribution of diacylglycerols. Diacylglycerols are higher in olive-derived oils as compared to seed oils [1], especially in OPOs because of the hydrolysis occurring during storage of olive pomace in ponds [30]. Refining removes free fatty acids but not the partial glycerides (diacylglycerols and monoacylglycerols), which remain in refined OPOs [31]. 

The fatty acid composition of the oils tested was within the limits established for OPOs, with oleic acid as the major fatty acid (70.36–73.29%), followed by palmitic acid (11.10–12.02%) and linoleic acid (9.19–11.46%). *Trans* fatty acids were below 0.35%, much lower than values previously reported in other OPOs (1.1%) [7].

Regarding the contents in bioactive compounds, tocopherols ranged from 310 to 496 mg/kg, which is usual for OPOs, even though the regulated maximum is 200 mg/kg [10]. The contents of phytosterols in the oils were higher than 2515 mg/kg and, as expected, over the levels typical of olive oils [10]. Contents of triterpenic acids, specifically oleanolic acid, were above 200 mg/kg. Crude olive-pomace oils normally show higher amounts of oleanolic acid, but refining conditions determine the final contents [11]. It should be noted that the elimination of triterpenic acids is a legal requirement since turbidity in refined oils is caused by their precipitation at room temperature, together with waxes and saturated triglycerides. Such compounds must be eliminated in the refining process to ensure that they are not deposited over time on the bottom of the container, which would result in an oil not complying with one of the quality characteristics required for Olive Oils and Olive Pomace Oils, i.e., the appearance, which must be limpid. Likewise, refining also affects squalene contents. As can be observed, very variable amounts of squalene were found, ranging from 387 to 3699 mg/kg. However, alcoholic compounds showed a narrower range of variability. Thus, values for aliphatic alcohols were between 1815 and 2136 mg/kg and values for erythrodiol were between 585 and 660 mg/kg. Overall, the levels of bioactive compounds depend on those in the initial crude oils and on the conditions used in the different stages of the refining process, in which considerable losses may occur. Thus, triterpenic acids as well as sterols and alcohols decrease during neutralization and, in addition, alcohols and squalene are partially distilled during deodorization [11].

### 3.2. Thermoxidation Assay

The modifications in the OPOs under thermoxidation conditions were examined in both the saponifiable, glyceridic fraction and the minor, unsaponifiable fraction. 

Table 2 shows the results obtained for the total content and proportions of polar compounds in OPOs thermoxidized for 20 h.

It is important to note that one of the advantages of the thermoxidation assay applied was its high reproducibility. This is achieved mainly by using standard tubes and a single heating block that allows for applying the same temperature, one of the most important variables in the frying process, for all samples [23]. In frying experiments, the results obtained are often more variable between different fryers, normally with less rigorous specifications in temperature operation conditions [32]. 

All OPOs exceeded the limit of 25% polar compounds at the end of the assay. This is the limit of rejection for human consumption established in most countries where alteration of frying fats and oils is regulated [33]. No changes in hydrolytic products (diacylglycerols, monoacylglycerols, and free fatty acids) were observed and the most abundant compounds formed were dimers and higher oligomers.

**Figure 1:**
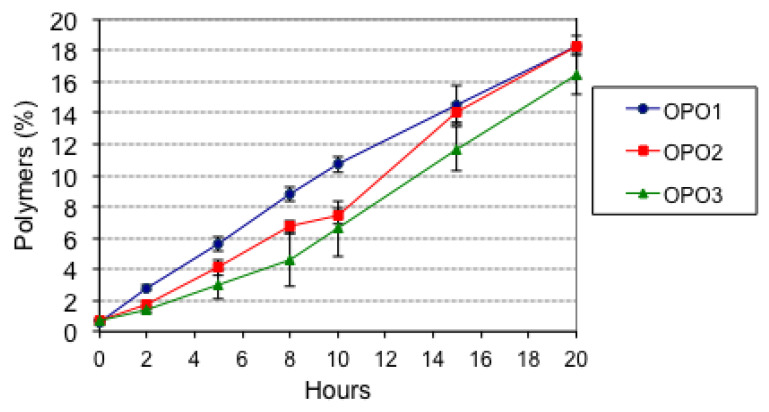
Formation of polymers in olive-pomace oils throughout thermoxidation.

Figure 1 shows the increase of polymers (sum of dimers and higher oligomers) throughout thermoxidation. 

In those countries where frying regulations include polymers as alteration measurement, their limits are established of 10–16% [33]. Direct measurement of polymers is an excellent analytical tool to evaluate oil degradation during thermoxidation and frying since polymers are the compounds mostly formed. As can be observed, OPO1 reached 10.7% polymers at 10 h while OPO2 and OPO3 did so between 10–15 h.

The results obtained for fatty acid composition throughout thermoxidation are shown in Table 3.

Changes in fatty acid composition also reflected the progress of alteration since a relative proportional increase in saturated acids and decrease in polyunsaturated acids (C18:2 and C18:3) were observed. Thus, for example, in OPO3, C18:2 decreased from 9.55% to 6.59% and C18:3 from 0.69% to 0.33% after 20 h. Furthermore, the real losses of polyunsaturated acids, due to formation of oxidized and polymeric compounds, are more pronounced and can be calculated by keeping constant the proportion of saturated acids [34]. As to *trans* fatty acids, a total level lower than 0.7% was found for all OPOs at the end of the assay. 

Table 4 and Table 5 show changes in sterols and other bioactive compounds throughout thermoxidation. 

As in all olive-derived oils, β-sitosterol was by far the most abundant sterol in all OPOs, accounting for over 85% of total sterols. Regarding total sterols, the amounts remaining after 20 h of thermoxidation were over 74%. Oxidation of sterols during oil heating has been reported [35,36] and polymerization reactions can also occur [37]. As can be observed, the results of sterols found in very small amounts showed high standard deviations and, hence, changes throughout thermoxidation were not generally significant. Therefore, overall results obtained also showed that there were no significant differences in the distribution of sterol classes throughout the assay, that is, all sterols were similarly affected by thermoxidation reactions. 

The most relevant losses were found in tocopherols, due to their rapid degradation at high temperatures and antioxidant action [38,39,40,41], and in squalene, which is very prone to oxidation due to its high number of double bonds [42] and which may also exert a combined action with tocopherols as secondary antioxidants [43]. Nevertheless, considerable amounts of squalene remained at the end of the thermoxidation assay, especially in OPO2, which started with the highest levels and kept over 1000 mg/kg at the end of the experiment. Aliphatic alcohols and erythrodiol were the compounds less affected by thermoxidation, whereas oleanolic acid decreased in a variable range, between 36% and 71%.

**Figure 2:**
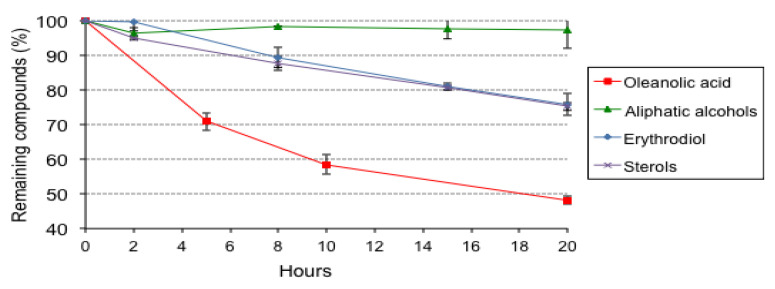
Mean values for remaining percentages of oleanolic acid, aliphatic alcohols, erythrodiol, and sterols in the three OPOs throughout thermoxidation assays.

Figure 2 illustrates the losses of those bioactive compounds that remained at levels close or over 50% throughout thermoxidation, considering all OPOs.

Regardless of the initial levels, Figure 2 shows that percentage losses of oleanolic acid, aliphatic alcohols, erythrodiol, and sterols were similar in all oils. As already commented, the alteration limit for human consumption was far surpassed after 20 h. Considering that levels of polymers should not exceed 10–16% [33] and that such levels were achieved between 10–15 h (Figure 1), Figure 2 illustrates that the remaining levels of oleanolic acid, erythrodiol, and sterols were then generally higher than at the end of the experiments.

### 3.3. Discontinuous Frying 

Three frying operations were carried out using the frozen foods selected, i.e., prefried French fries, chicken croquettes, and prefried, battered chicken nuggets, using OPO1. Results included in this work correspond to the fourth frying operation. As expected, after frying, all foods decreased in moisture content and showed an increment in lipid content that depended on the food characteristics, differing greatly in the frozen foods selected. The higher lipid increments were found in the prefried potatoes (from 11.2 to 23.9%), followed by croquettes (from 26.6 to 35.9%) and nuggets (from 32.9 to 40.1%). It is well-known that food surface structure and composition, moisture, lipid content, product shape and surface-to-weight ratio, porosity, and pre-frying treatment are relevant variables affecting the final composition of the finished fried product [44]. 

Table 6 shows the total content of polar compounds and their distribution by molecular size in initial and final OPO and initial and fried food products. Total polar compounds in frying oils were twice as high after the fourth frying operation and similar levels were found regardless of the product fried, about 14%, far from the limit of rejection (25% polar compounds). The increase in polar compounds was essentially due to the formation of oxidized and polymeric compounds. Hydrolytic products (diacylglycerols, monoacylglycerols, and free fatty acids) remained at low levels, as it occurred in the thermoxidation study (Table 2), although a slight increase in diacylglycerols was observed in oils used to fry croquettes and nuggets. A remarkable decrease in polar compounds was found in the French fries as compared to the initial prefried product, in which the level was over the limit for rejection. Therefore, the high lipid exchange that took place improved greatly the quality of the final French fries. 

Table 7 shows fatty acid composition in initial and final OPOs and initial and fried food products. 

After four frying operations, the fatty acid compositions of the oils did not differ significantly from the initial values while remarkable differences were found in frozen foods after frying. This was especially reflected in the significant increase in oleic and decrease in linoleic acid contents, as a result of frying oil absorption and lipid exchange. Based upon the contents of fatty acids, lipid exchange can be calculated with high accuracy when the differences in the content of some fatty acids between the food lipids and the frying oil are over 30% [45]. Therefore, the proportion of frying oil in the fried foods, as estimated from changes in percentages of oleic acid, showed that the lipid content in the fried products coming from the frying oil were 90.6%, 49.0%, and 51.3% in French fries, chicken croquettes, and chicken nuggets, respectively. This means that the lipid quality and composition of the final fried products, especially in the case of French fries, were mostly dependent on the quality and composition of the oil used for frying. The high extent of oil exchange in prefried French fries is due to the fact that all lipids in these products come from the oil used for prefrying, which is mainly located on the surface and, thus, it is readily released into the frying oil [44]. Chicken nuggets were also prefried products, but their coverage with batter normally diminishes the lipid exchange with the frying oil, as it also occurs with bread coverage [44,45,46,47,48].

The contents in sterols and the other bioactive compounds in OPOs and foods initially and after frying are shown in the Table 8 and Table 9. 

In general, the total contents of sterols expressed as mg/kg on food lipids were lower in the fried than in the initial products because of the contribution of the frying oil, which contained lower levels. However, it is important to bear in mind that the lipid content was always higher in the foods after frying and, consequently, also the sterol content expressed on food weight. In fact, expressed in kilogram of fried food, sterol contents after frying were only lower in the case of croquettes (from 929 to 819 mg/kg food) and higher in the case of nuggets (from 1154 to 1217 mg/kg food) and especially in French fries (444 to 647 mg/kg food). The results obtained in French fries were consistent with those reported in other studies using sunflower, cottonseed, palm, and olive oils for frying French fries [21,49,50,51,52]. In the present study, all fried products were enriched predominantly in β-sitosterol, the most abundant sterol in OPO and in all olive-derived oils, while cholesterol significantly decreased in the products containing chicken. It is evident that lower amounts of cholesterol could have also been found after frying the products containing chicken with any other vegetable oil.

Results in Table 9 showed great losses of tocopherols in OPO after the fourth frying operation, as already observed in the thermoxidation assay (Table 5). The fried products also showed lower levels after frying when they contained tocopherols initially. The tocopherol content of French fries has been evaluated in a number of studies [44,49,52,53,54] and it is generally agreed that, regardless of the type or quality of the frying oil, no preferential adsorption of tocopherol on the food surface takes place. 

Squalene was absent or in negligible amounts in the initial foods and, since about 60–80% remained in the OPO after the fourth frying operation, its content in the fried products was relatively high as a result of oil absorption. Incorporation of squalene in fried products is nutritionally positive since it is a bioactive compound with recognized anti-carcinogenic, antioxidant, hypocholesterolemic, and detoxifying activities [55,56]. The results obtained in the present study agreed with those reported by Kalogeropoulos and Andrikopoulos [57] in fried potatoes and those published by de Alzaa et al. [22] in nuggets, in both studies using virgin olive oil. In practical terms, based upon the data obtained in the present study, one serving (200 g) of French fries, croquettes, or nuggets would supply about 34, 36, or 37 mg of squalene, respectively. It is important to note that enrichment with squalene could have been much greater since the oil used in this study only contained about 1000 mg/kg, whereas amounts as high as 6000 mg/kg can be found in OPOs [1].

After four frying operations, the oil contained practically the same levels of triterpenic alcohols and aliphatic alcohols, while triterpenic acids showed slight significant losses. These results were consistent with those found throughout the thermoxidation study (Table 5). Since these oil components were absent or present in very low amounts in the initial foods, the extent of their incorporation into the fried products reflected that the highest lipid exchange occurred in French fries, as already estimated according to changes in oleic acid (Table 7). Triterpenic and aliphatic alcohols are characteristic of OPOs and have shown health-promoting effects [14,15,16,17,18,19,20]. In the case of triterpenic alcohols, one serving (200 g) of French fries, croquettes, or nuggets would supply 30.5, 23.0, and 22.5 mg of triterpenic alcohols, respectively. Particularly relevant was the content of aliphatic alcohols in fried foods since one serving of French fries, croquettes, or nuggets would supply 92.7, 81.1, and 88.2 mg aliphatic alcohols, respectively. These are markedly high amounts considering that only 5–20 mg per day of mixed C24–C34 alcohols have been suggested to improve the lipid profile in humans [19]. 

## 4. Conclusions

The results obtained in this study demonstrated for the first time that the characteristic minor, bioactive compounds of olive-pomace oil present high thermoxidative stability, especially triterpenic and aliphatic alcohols. Thus, in samples with total oil alteration levels of approximately 30% polar compounds, over the regulated limit for human consumption, no significant losses of triterpenic alcohols and aliphatic alcohols were found and the remaining levels of sterols and triterpenic acids were as high as 75–80% and about 50%, respectively. In contrast, significant losses of squalene (71–84%) were observed, although the remaining amounts in the olive-pomace oil were still considerably high. 

The incorporation of olive-pomace oil into the food products studied due to oil absorption increased their lipid content, as expected, but had positive nutritional effects, i.e., the increase in oleic acid and in minor bioactive compounds. Among the bioactive compounds in olive-pomace oil, those absent or present in relatively low amounts in the initial foods were found in relatively high amounts in the fried products, i.e., squalene, aliphatic, and triterpenic alcohols. 

## Figures and Tables

**Table 1:** Characterization parameters of unused olive-pomace oils.

	OPO1	OPO2	OPO3
Fatty Acids (wt%) C16:0	12.02 ± 0.01c	11.10 ± 0.00a	11.42 ± 0.01b
C16:1	0.87 ± 0.00c	0.72 ± 0.00a	0.83 ± 0.01b
C18:0	2.94 ± 0.01b	2.75 ± 0.01a	2.84 ± 0.01b
C18:1	70.36 ± 0.01a	73.29 ± 0.04c	72.69 ± 0.01b
C18:2	11.46 ± 0.01c	9.19 ± 0.01a	9.55 ± 0.01b
C18:3	0.72 ± 0.02c	0.65 ± 0.00a	0.69 ± 0.01b
C20:0	0.46 ± 0.01b	0.40 ± 0.00a	0.44 ± 0.02b
C20:1	0.31 ± 0.01a	0.31 ± 0.00a	0.35 ± 0.00b
C22:0	0.12 ± 0.10a	0.18 ± 0.01a	0.19 ± 0.00b
C24:0	nd	0.07 ± 0.06a	0.07 ± 0.00a
Others	0.24 ± 0.01a	1.07 ± 0.00c	0.60 ± 0.00b
*Trans* C18:1	0.21 ± 0.00a	0.23 ± 0.00b	0.27 ± 0.00c
*Trans* C18:2	nd	0.06 ± 0.00a	0.07 ± 0.00a
*Σ Trans*	0.21 ± 0.00a	0.29 ± 0.00b	0.34 ± 0.00c
Polar Compounds (wt%) Total	7.6 ± 0.2a	10.6 ± 2.1b	10.1 ± 0.4b
Oligomers	nd	nd	nd
Dimers	0.6 ± 0.1a	0.8 ± 0.0a	0.7 ± 0.1a
Oxidized Triacylglycerols	0.9 ± 0.1a	1.3 ± 0.3a	1.3 ± 0.2a
Diacylglycerols	5.4 ± 0.1a	7.5 ± 1.6a	7.3 ± 0.3a
Monoacylglycerols	0.2 ± 0.0a	0.2 ± 0.0a	0.2 ± 0.0a
Free Fatty Acids *	0.5 ± 0.0a	0.8 ± 0.1b	0.6 ± 0.1a
Bioactive compounds (mg/kg)			
Tocopherols	496 ± 13c	344 ± 6b	310 ± 4a
Squalene	1062 ± 32b	3699 ± 58c	387 ± 13a
Sterols	2515 ± 26a	3021 ± 23b	3438 ± 17c
Aliphatic alcohols	1875 ± 56a	1815 ± 5a	2136 ± 33b
Erythrodiol	616 ± 13ab	585 ± 39a	660 ± 18b
Oleanolic Acid	201 ± 10a	425 ± 45b	358 ± 52b

Results are expressed as means ± SD (*n* = 3). Different letters in the same row indicate significant differences according to Tukey´s test at *p* < 0.05; * it also includes the polar unsaponifiable matter; nd: not detected.

**Table 2:** Polar compound content and distribution in olive-pomace oils thermoxidized for 20 h.

Polar Compounds (wt%)	OPO1	OPO2	OPO3
Total	33.1 ± 3.5a	34.8 ± 1.7a	38.0 ± 2.3a
Oligomers	7.4 ± 1.1a	8.3 ± 0.3a	8.8 ± 0.5a
Dimers	10.3 ± 0.4a	9.0 ± 0.7a	10.2 ± 0.7a
Oxidized Triacylglycerols	9.5 ± 2.3a	11.6 ± 0.4a	11.2 ± 0.4a
Diacylglycerols	5.2 ± 0.2a	5.3 ± 0.3a	7.0 ± 0.5b
Monoacylglycerols	0.1 ± 0.1a	0.2 ± 0.0a	0.2 ± 0.0a
Free Fatty Acids *	0.4 ± 0.0a	0.5 ± 0.0a	0.5 ± 0.1a

Results are expressed as means ± SD (*n* = 3). Different letters in the same row indicate significant differences according to Tukey´s test at *p* < 0.05; * it also includes the polar unsaponifiable matter.

**Table 3:** Fatty acid composition (wt%) in olive-pomace oils throughout thermoxidation.

Oil	0 h	2 h	8 h	15 h	20 h
OPO1					
C16:0	12.02 ± 0.01a	12.84 ± 0.14b	13.06 ± 0.02b	13.37 ± 0.08c	13.64 ± 0.14d
C16:1	0.87 ± 0.00a	0.93 ± 0.02b	0.97 ± 0.00c	0.97 ± 0.00c	0.97 ± 0.01c
C18:0	2.94 ± 0.01bc	2.83 ± 0.02a	2.89 ± 0.02b	2.96 ± 0.03cd	3.01 ± 0.04d
C18:1	70.36 ± 0.01d	69.76 ± 0.27c	69.53 ± 0.09bc	69.35 ± 0.04ab	69.15 ± 0.11a
C18:2	11.46 ± 0.01d	10.93 ± 0.37d	10.01 ± 0.06c	9.09 ± 0.25b	8.34 ± 0.41a
C18:3	0.72 ± 0.01d	0.59 ± 0.04c	0.49 ± 0.01b	0.40 ± 0.02a	0.34 ± 0.03a
C20:0	nd	0.45 ± 0.01a	0.51 ± 0.00b	0.51 ± 0.00b	0.53 ± 0.00c
C20:1	0.31 ± 0.00a	0.31 ± 0.01a	0.36 ± 0.01b	0.37 ± 0.02bc	0.38 ± 0.01c
C22:0	0.12 ± 0.10a	0.18 ± 0.01a	0.21 ± 0.01a	0.21 ± 0.01a	0.23 ± 0.01a
C24:0	nd	nd	0.10 ± 0.02b	0.09 ± 0.00b	0.09 ± 0.00b
Others	0.24 ± 0.01a	1.10 ± 0.52b	1.40 ± 0.10bc	2.10 ± 0.17cd	2.67 ± 0.32d
*t*C18:1	0.21 ± 0.00a	0.37 ± 0.02b	0.40 ± 0.01b	0.50 ± 0.02c	0.57 ± 0.04d
*t*C18:2	nd	nd	0.07 ± 0.00b	0.08 ± 0.01bc	0.08 ± 0.01c
*∑trans*	0.21 ± 0.00a	0.37 ± 0.02b	0.47 ± 0.01c	0.58 ± 0.03d	0.66 ± 0.03e
OPO2					
C16:0	11.10 ± 0.00a	11.26 ± 0.03b	11.68 ± 0.05c	12.17 ± 0.05d	12.27 ± 0.06d
C16:1	0.72 ± 0.00a	0.73 ± 0.00b	0.74 ± 0.01c	0.75 ± 0.00c	0.73 ± 0.00b
C18:0	2.75 ± 0.01a	2.78 ± 0.02a	2.88 ± 0.02b	3.00 ± 0.02c	3.02 ± 0.03c
C18:1	73.29 ± 0.04b	73.74 ± 0.04c	74.15 ± 0.03d	74.47 ± 0.01e	73.02 ± 0.10a
C18:2	9.19 ± 0.01e	8.97 ± 0.09d	7.96 ± 0.10c	6.91 ± 0.09b	6.07 ± 0.09a
C18:3	0.65 ± 0.00e	0.61 ± 0.02d	0.48 ± 0.01c	0.36 ± 0.01b	0.28 ± 0.01a
C20:0	0.40 ± 0.00a	0.40 ± 0.00a	0.41 ± 0.01a	0.44 ± 0.00b	0.46 ± 0.01b
C20:1	0.31 ± 0.00a	0.32 ± 0.01a	0.37 ± 0.00b	0.38 ± 0.00c	0.37 ± 0.00b
C22:0	0.18 ± 0.01ab	0.17 ± 0.00a	0.19 ± 0.01abc	0.21 ± 0.01c	0.20 ± 0.02bc
C24:0	0.07 ± 0.00a	0.06 ± 0.00a	0.07 ± 0.00a	0.08 ± 0.00b	0.08 ± 0.00b
Others	1.07 ± 0.06b	0.63 ± 0.06a	0.67 ± 0.06a	0.70 ± 0.00a	2.83 ± 0.06c
*t*C18:1	0.23 ± 0.00a	0.25 ± 0.01a	0.35 ± 0.02b	0.48 ± 0.01c	0.56 ± 0.03d
*t*C18:2	0.06 ± 0.00a	0.06 ± 0.00a	0.06 ± 0.01ab	0.08 ± 0.01c	0.07 ± 0.00bc
*∑trans*	0.29 ± 0.00a	0.31 ± 0.01a	0.41 ± 0.03b	0.55 ± 0.01c	0.63 ± 0.02d
OPO3					
C16:0	11.42 ± 0.01a	11.52 ± 0.02a	11.81 ± 0.13ab	12.09 ± 0.36bc	12.52 ± 0.08c
C16:1	0.83 ± 0.01a	0.85 ± 0.01ab	0.85 ± 0.00ab	0.85 ± 0.01b	0.84 ± 0.01ab
C18:0	2.84 ± 0.01a	2.84 ± 0.01a	2.92 ± 0.04ab	2.99 ± 0.09bc	3.09 ± 0.02c
C18:1	72.69 ± 0.01b	72.81 ± 0.01b	73.13 ± 0.10c	73.30 ± 0.17c	72.30 ± 0.17a
C18:2	9.55 ± 0.01c	9.43 ± 0.05c	8.68 ± 0.27bc	8.10 ± 0.71b	6.59 ± 0.22a
C18:3	0.69 ± 0.01d	0.67 ± 0.01cd	0.56 ± 0.04bc	0.49 ± 0.08b	0.33 ± 0.02a
C20:0	0.44 ± 0.02a	0.47 ± 0.00ab	0.48 ± 0.00b	0.49 ± 0.02b	0.50 ± 0.00b
C20:1	0.35 ± 0.00a	0.35 ± 0.01a	0.38 ± 0.01b	0.39 ± 0.01b	0.38 ± 0.01b
C22:0	0.19 ± 0.00a	0.19 ± 0.01a	0.19 ± 0.00a	0.20 ± 0.01a	0.23 ± 0.01b
C24:0	0.07 ± 0.00a	0.07 ± 0.00a	0.08 ± 0.00b	0.08 ± 0.01b	0.09 ± 0.01b
Others	0.60 ± 0.00a	0.47 ± 0.06a	0.50 ± 0.00a	0.53 ± 0.06a	2.53 ± 0.32b
*t*C18:1	0.27 ± 0.00a	0.28 ± 0.01a	0.35 ± 0.03ab	0.41 ± 0.08b	0.55 ± 0.02c
*t*C18:2	0.07 ± 0.00a	0.07 ± 0.00a	0.07 ± 0.00a	0.07 ± 0.01a	0.07 ± 0.00a
*∑trans*	0.34 ± 0.00a	0.35 ± 0.01a	0.42 ± 0.03ab	0.49 ± 0.09b	0.62 ± 0.02c

Results are expressed as means ± SD (*n* = 3). Different letters in the same row indicate significant differences according to Tukey´s test at *p* < 0.05. nd, not detected

**Table 4:** Total content and composition of sterols in olive-pomace oils throughout thermoxidation.

Sterol (% on Total)	0 h	2 h	8 h	15 h	20 h
OPO1					
Chol	0.34 ± 0.03a	0.63 ± 0.19a	1.41 ±1.06a	2.18 ± 2.04a	1.86 ± 2.80a
Camp	3.30 ± 0.04a	3.76 ± 0.14a	3.26 ±0.17a	3.20 ± 0.05a	3.83 ± 0.85a
Stigm	1.55 ± 0.03a	2.88 ± 0.79b	1.15 ±0.06a	1.14 ± 0.02a	1.32 ± 0.25a
Δ7-Camp	1.20 ± 0.00a	1.33 ± 0.00b	1.30 ±0.03b	1.32 ± 0.02b	1.50 ± 0.00c
Cler	0.00 ± 0.07a	0.08 ±0.07a	0.11 ±0.03a	0.00 ± 0.03a	0.05 ± 0.29a
β-Sito	85.46 ± 0.06a	85.09 ± 0.20a	86.14 ± 0.10a	85.86 ± 0.00a	85.85 ± 0.09a
Sitos	2.11 ± 0.04a	3.20 ± 1.26ab	2.83 ± 1.03ab	2.96 ± 1.97ab	3.44 ± 2.32b
Δ5-Aven	1.66 ± 0.07b	1.01 ± 0.03ab	1.43 ± 0.22ab	1.19 ± 0.36a	1.44 ± 0.58ab
Δ5,24-Sti	2.07 ± 0.02a	1.12 ± 0.08a	1.71 ± 0.07a	1.72 ± 0.04a	1.94 ± 0.28a
Δ7-Stigmn	0.62 ± 0.02a	0.64 ± 0.85b	0.45 ± 0.15a	0.36 ± 0.01a	0.48 ± 0.30a
Δ7-Aven	0.30 ± 0.03a	0.00 ± 0.87a	0.00 ± 0.04a	0.10 ± 0.03a	0.00 ± 0.16a
Total (mg/kg)	2515 ± 26a	2387 ± 34b	2155 ± 34c	2045 ± 56 d	1914 ± 25 e
OPO2					
Chol	1.08 ± 1.44a	0.30 ± 0.05a	0.27 ± 0.08a	0.34 ± 0.04a	0.30 ± 0.04a
Camp	2.99 ± 0.03a	2.98 ± 0.00a	2.94 ± 0.00a	2.98 ± 0.00a	2.98 ± 0.00a
Stigm	1.31 ± 0.08b	1.28 ± 0.02ab	1.24 ± 0.05ab	1.22 ± 0.00a	1.21 ± 0.02a
Δ7-Camp	0.47 ± 0.04a	0.48 ± 0.02a	0.45 ± 0.01a	0.43 ± 0.02a	0.43 ± 0.03a
Cler	0.02 ± 0.01a	0.03 ± 0.03a	0.04 ± 0.01a	0.05 ±0.01a	0.05 ± 0.01a
β-Sito	86.16 ± 0.04a	87.56 ± 0.05a	87.67 ± 0.07a	87.87 ±0.09a	87.78 ± 0.09a
Sitos	2.89 ± 1.36b	2.66 ± 0.17a	2.89 ± 0.31ab	3.08 ± 0.30b	3.03 ± 0.20b
Δ5-Aven	3.31 ± 0.08a	3.08 ± 0.13a	2.98 ± 0.15a	2.82± 0.08a	2.80 ± 0.07b
Δ5,24-Sti	1.05 ±0.04b	0.93 ± 0.23ab	0.95 ± 0.28ab	0.79 ± 0.28a	0.90 ± 0.06ab
Δ7-Stigmn	0.36 ± 0.04b	0.44 ± 0.03b	0.34 ± 0.07ab	0.29 ± 0.06a	0.32 ± 0.09ab
Δ7-Aven	0.27 ± 0.02a	0.27 ± 0.08a	0.24 ± 0.04a	0.14 ± 0.02a	0.19 ± 0.07a
Total (mg/kg)	3021 ± 23a	2892 ± 48b	2643 ± 17c	2416 ± 21 d	2235 ± 23 e
OPO3					
Chol	0.23 ± 0.01a	0.21 ± 0.04a	0.27 ± 0.02a	0.28 ± 0.09a	0.21 ± 0.03a
Camp	2.92 ± 0.04a	2.92 ± 0.04a	2.94 ± 0.15a	2.93 ± 0.11a	2.88 ± 0.10a
Stigm	1.42 ± 0.03b	1.36 ± 0.01ab	1.32 ± 0.05ab	1.34 ± 0.05ab	1.32 ± 0.03a
Δ7-Camp	0.81 ± 0.04a	0.83 ± 0.05a	0.79 ± 0.05a	0.77 ± 0.05a	0.71 ± 0.00a
Cler	0.01 ± 0.03a	0.01 ± 0.02a	0.02 ± 0.05a	0.03 ± 0.08a	0.03 ± 0.05a
β-Sito	87.11 ± 0.31a	87.77 ± 0.19a	87.43 ± 0.59a	87.90 ± 0.38a	88.11 ± 0.38a
Sitos	2.92 ± 0.04a	2.58 ± 0.10a	3.04 ± 0.81a	2.97 ± 0.55a	3.18 ± 0.60a
Δ5-Aven	1.82 ± 0.05a	1.74 ± 0.08a	1.76 ± 0.27a	1.60 ± 0.23a	1.54 ± 0.25a
Δ5,24-Sti	2.10 ± 0.05c	1.92 ± 0.11bc	1.77 ± 0.12abc	1.62 ± 0.17ab	1.43 ± 0.15a
Δ7-Stigmn	0.46 ± 0.12a	0.40 ± 0.08a	0.39 ± 0.15a	0.33 ± 0.04a	0.36 ± 0.12a
Δ7-Aven	0.14 ± 0.07a	0.20 ± 0.03a	0.20 ± 0.06a	0.18 ± 0.04a	0.23 ± 0.13a
Total (mg/kg)	3438 ± 17a	3252 ± 25b	3084 ± 56c	2789 ± 36 d	2606 ± 13 e

Results are expressed as means ± SD (*n* = 3). Different letters in the same row indicate significant differences according to Tukey´s test at *p* < 0.05. Abbreviations: Chol, Cholesterol; Camp, Campesterol; Stigm, Stigmasterol; Δ7-Camp, Δ7-Campesterol; Cler, Clerosterol; β-Sito, β-Sitosterol; Sitos, Sitostanol; Δ5-Aven, Δ5-Avenasterol; Δ5,24-Sti, Δ5,24-Stigmastadienol; Δ7-Stigmn, Δ7-Stigmastenol; Δ7-Ave, Δ7-Avenasterol.

**Table 5:** Bioactive compounds in olive-pomace oils throughout thermoxidation.

	Tocopherols (mg/kg)	Squalene (mg/kg)	Aliphatic Alcohols (mg/kg)	Erythrodiol (mg/kg)	Oleanolic Acid (mg/kg)
OPO1					
0 h	496 ± 13a	1062 ± 32a	1875 ± 56a	616 ± 13a	210 ± 10a
2 h	215 ± 13b	525 ± 21b	n.a.	n.a.	n.a.
5 h	45 ± 3c	407 ± 20c	2066 ± 152a	552 ± 73ab	142 ± 8b
8 h	9 ± 1d	342 ± 10d	n.a.	n.a.	n.a.
10 h	10 ± 5d	302 ± 10de	2177 ± 465a	532 ± 14ab	119 ± 9c
15 h	0	266 ± 13e	1895 ± 111a	505 ± 3b	n.a.
20 h	0	166 ± 24f	1938 ± 16a	487 ± 18b	105 ± 12c
OPO2					
0 h	344 ± 6a	3699 ± 58a	1815 ± 5a	585 ± 39a	425 ± 45a
2 h	167 ± 67b	3363 ± 21b	1788 ± 19ab	594 ± 16a	n.a.
5 h	23 ± 0c	n.a.	n.a.	n.a.	380 ± 43a
8 h	25 ± 1c	2172 ± 43c	1788 ± 32ab	525 ± 8b	n.a.
10 h	26 ± 1c	n.a.	n.a.	n.a.	287 ± 5b
15 h	29 ± 2c	1412 ± 32d	1766 ± 30ab	467 ± 15c	n.a.
20 h	23 ± 2c	1083 ± 23e	1738 ± 20b	426 ± 1c	271 ± 8b
OPO3					
0 h	310 ± 4a	491 ± 28a	2136 ± 33a	660 ± 18a	358 ± 52a
2 h	283 ± 25a	387 ± 13b	2084 ± 22ab	646 ± 10ab	n.a.
5 h	219 ± 20b	n.a.	n.a.	n.a.	189 ± 9b
8 h	47 ± 13c	267 ± 18c	2104 ± 47ab	608 ± 25b	n.a.
10 h	43 ± 4c	n.a.	n.a.	n.a.	175 ± 20c
15 h	40 ± 4c	166 ± 13d	2032 ± 30ab	536 ± 12c	n.a.
20 h	37 ± 5c	116 ± 13e	1989 ± 78b	499 ± 4c	102 ± 28c

Results are expressed as means ± SD (*n* = 3). Different letters in the same row indicate significant differences according to Tukey´s test at *p* < 0.05; n.a.: not analyzed

**Table 6:** Polar compound content and distribution in initial and final olive-pomace oils and in initial and fried food lipids.

Polar Compounds (wt%)	Total	Oligomers	Dimers	Oxidized Triacylglycerols	Diacyl Glycerols	Monoacyl Glycerols	Free Fatty Acids
Initial OPO	7.6 ± 0.4	nd	0.6 ± 0.1	0.9 ± 0.1	5.4 ± 0.2	0.2 ± 0.0	0.5 ± 0.0
French fries							
Initial	28.3 ± 02b	6.9 ± 0.1b	9.3 ± 0.1b	8.6 ± 0.1b	2.7 ± 0.1a	nd	0.8 ± 0.0b
Fried	14.2 ± 0.2a	1.0 ± 0.1a	3.6 ± 0.1a	3.9 ± 0.4a	4.9 ± 0.3b	0.1 ± 0.0a	0.6 ± 0.0a
Final OPO	14.5 ± 0.3a	0.9 ± 0.0a	3.7 ± 0.1a	3.9 ± 0.1a	5.3 ± 0.2b	0.2 ± 0.0b	0.6 ± 0.0a
Croquettes							
Initial	8.1 ± 0.4a	1.5 ± 0.2b	0.8 ± 0.1a	4.6 ± 0.2b	1.2 ± 0.0a	nd	1.1 ± 0.28c
Fried	12.0 ± 2.0b	0.8 ± 0.2a	2.6 ± 0.6b	4.5 ± 0.8b	3.3 ± 0.4b	0.1±0.01a	0.75 ± 0.04b
Final OPO	13.8 ± 0.9b	0.6 ± 0.1a	2.5 ± 0.3b	2.6 ± 0.4a	7.6 ± 0.3c	0.16±0.05a	0.47 ± 0.12a
Nuggets							
Initial	8.6 ± 1.3a	nd	2.6 ± 0.6a	3.3 ± 0.4a	1.7 ± 0.4a	nd	1 ± 0.1b
Fried	10.3 ± 1.44b	0.4 ± 0.08a	1.8 ± 0.32a	2.3 ± 0.29a	4 ± 0.89b	0.7 ± 0.19b	1 ± 0.32b
Final OPO	13.4 ± 2.2b	0.5 ± 0.2a	2.2 ± 0.6a	2.3 ± 0.8a	7.9 ± 0.6c	0.1 ± 0.01a	0.3 ± 0.1a

Results are expressed as means ± SD (*n* = 3). Different letters in the same column within the same food frying operations (initial food, final food, and final OPO) indicate significant differences according to Tukey´s test at *p* < 0.05; nd: not detected.

**Table 7:** Fatty acid composition in initial and final olive-pomace oils and in initial and fried food lipids.

	OPO	French Fries			Croquettes			Nuggets		
Fatty Acid (%)	Initial	Initial	Fried	Final OPO	Initial	Fried	Final OPO	Initial	Fried	Final OPO
C16:0	12.02 ± 0.01	11.55 ± 0.03a	11.99 ± 0.02b	12.19 ± 0.02c	14.44 ± 0.02c	12.83 ± 0.14b	12.19 ± 0.02a	16.9 ± 0.02b	17.54 ± 0.1b	12.21 ± 0.09a
C16:1	0.87 ± 0.00	0.13 ± 0.01a	0.84 ± 0.01b	0.89 ± 0.00c	1.55 ± 0.00c	1.08 ± 0.03b	0.9 ± 0.01a	3.73 ± 0.02b	2.16 ± 1.85b	0.9 ± 0.02a
C17:0	0.09 ± 0.00	nd	0.09 ± 0.00a	0.09 ± 0.00a	0.16 ± 0.00c	0.12 ± 0.01b	0.10 ± 0.01a	0.09 ± 0.01a	0.11 ± 0.01a	0.09 ± 0.01a
C17:1	0.15 ± 0.01	nd	0.14 ± 0.01a	0.15 ± 0.00a	0.14 ± 0.00ab	0.13 ± 0.01a	0.15 ± 0.01b	0.07 ± 0.01a	0.13 ± 0.01b	0.15 ± 0.01b
C18:0	2.94 ± 0.01	3.43 ± 0.01b	3.04 ± 0.00a	3.05 ± 0.01a	6.31 ± 0.01c	4.52 ± 0.17b	3.05 ± 0.01a	4.77 ± 0.01c	4.23 ± 0.08b	3.02 ± 0.09a
C18:1	70.41 ± 0.07	42.52 ± 0.06a	67.79 ± 0.1b	70.13 ± 0.05c	38.42 ± 0.02a	54.1 ± 1.15b	70.3 ± 0.02c	43.73 ± 0.06a	57.44 ± 0.11b	69.91 ± 0.29c
C18:2	11.47 ± 0.02	40.62 ± 0.09c	13.43 ± 0.1b	10.85 ± 0.09a	35.25 ± 0.07c	23.95 ± 0.83b	10.69 ± 0.07a	27.18 ± 0.01c	13.81 ± 0.27b	11.12 ± 0.3a
C18:3	0.72 ± 0.01	0.24 ± 0.02a	0.67 ± 0.01c	0.60 ± 0.01b	0.45 ± 0.01a	0.54 ± 0.02b	0.61 ± 0.01c	0.60 ± 0.01a	0.76 ± 0.01b	0.60 ± 0.02a
C20:0	0.46 ± 0.01	0.24 ± 0.01a	0.47 ± 0.01b	0.50 ± 0.00c	0.22 ± 0.00a	0.37 ± 0.01b	0.50 ± 0.01c	0.23 ± 0.00a	0.37 ± 0.00b	0.50 ± 0.00c
C20:1	0.31 ± 0.00	0.17 ± 0.00a	0.35 ± 0.00b	0.36 ± 0.01b	0.49 ± 0.00b	0.39 ± 0.01a	0.37 ± 0.01a	0.34 ± 0.01a	0.39 ± 0.01c	0.37 ± 0.00b
C22:0	nd	0.54 ± 0.03c	0.22 ± 0.00b	0.20 ± 0.00a	0.36 ± 0.00c	0.01 ± 0.01a	0.19 ± 0.01b	0.04 ± 0.00a	0.03 ± 0.00a	0.20 ± 0.00b
C24:0	nd	0.19 ± 0.01b	0.09 ± 0.01a	0.08 ± 0.00a	0.12 ± 0.00c	0.11 ± 0b	0.08 ± 0.01a	0.12 ± 0.01c	0.06 ± 0.00a	0.08 ± 0.01b
Others	0.37 ± 0.09	0.22 ± 0.01a	0.59 ± 0.02b	0.63 ± 0.04b	1.8 ± 0.06c	1.57 ± 0.03b	0.57 ± 0.06a	1.84 ± 0.12b	2.66 ± 1.95b	0.57 ± 0.06a
*t*C18:1	0.21 ± 0.00	nd	0.24 ± 0.00a	0.24 ± 0.00a	0.23 ± 0.01a	0.22 ± 0.01a	0.26 ± 0.01b	0.20 ± 0.00a	0.27 ± 0.01b	0.27 ± 0.01b
*t*C18:2	nd	0.14 ± 0.01b	0.04 ± 0.00a	0.03 ± 0.01a	0.09 ± 0.00b	0.08 ± 0.01b	0.03 ± 0.00a	0.02 ± 0.01a	0.05 ± 0.01b	0.04 ± 0.00b
*∑trans*	0.21 ± 0.0 b	0.14 ± 0.01a	0.28 ± 0.01 b	0.27 ± 0.01b	0.32 ± 0.01b	0.30 ± 0.01ab	0.29 ± 0.01a	0.32 ± 0.01a	0.32 ± 0.01a	0.31 ± 0.01a

Results are expressed as means ± SD (*n* = 3). Different letters in the same row within the same food frying operations (initial food, final food, and final OPO) indicate significant differences according to Tukey´s test at *p* < 0.05; nd: not detected.

**Table 8:** Total content and composition of sterols in initial and final olive-pomace oils and in initial and fried food lipids.

Sterols	OPO	French Fries			Croquettes			Nuggets		
(% on Total)	Initial	Initial	Fried	Final OPO	Initial	Fried	Final OPO	Initial	Fried	Final OPO
Cholest	0.34 ± 0.02	0.59 ± 0.06a	0.40 ± 0.20a	0.55 ± 0.20a	36.95 ± 0.66c	21.86 ± 1.93b	0.50 ± 0.07a	61.20 ± 1.40c	55.53 ± 0.89b	0.54 ± 0.23a
Brassicast	0.11 ± 0.01	0.19 ± 0.08	nd	nd	nd	0.01 ± 0.05a	0.52 ± 0.90b	nd	0.16 ± 0.02a	0.07 ± 0.12a
Campest	3.30 ± 0.20	7.47 ± 0.07b	3.59 ± 0.60a	3.64 ± 0.60a	6.85 ± 0.01a	5.80 ± 0.05a	4.45 ± 2.09a	4.17 ± 0.14c	2.48 ± 0.01a	3.14 ± 0.05b
Campest	0.35 ± 0.02	0.94 ± 0.12	nd	nd	1.16 ± 0.02	0.71 ± 0.00	0.19 ± 0.02	0.52 ± 0.02b	0.39 ± 0.02a	nd
Stigmast	1.55 ± 0.08	7.48 ± 0.16b	1.77 ± 0.60a	1.84 ± 0.60a	4.24 ± 0.08c	3.14 ± 0.02b	1.08 ± 0.08a	2.57 ± 0.09c	0.58 ± 0.50a	1.3 ± 0.20b
Δ7-Campest	0.87 ± 0.04	2.08 ± 0.12b	0.25 ± 0.2a	0.25 ± 0.20a	0.97 ± 0.13b	0.80 ± 0.07b	0.10 ± 0.09a	0.81 ± 0.03b	0.17 ± 0.01a	nd
Δ5,23-Stigmastadienol	1.20 ± 0.06	1.28 ± 0.24a	1.28 ± 0.01a	1.30 ± 0.10a	0.37 ± 0.04a	0.77 ± 0.06b	1.12 ± 0.06c	0.27 ± 0.02a	0.42 ± 0.11b	1.16 ± 0.04c
Clerost	nd	2.83 ± 1.26	nd	nd	0.29 ± 0.41b	nd	0.03 ± 0.06a	0.03 ± 0.03a	0.16 ± 0.28a	nd
β-Sitost	85.46 ± 4.30	49.88 ± 1.91a	84.55 ± 2.80b	84.51 ± 1.20b	36.60 ±0.19a	56.84 ± 1.91b	85.72 ± 2.68c	21.33 ± 0.77a	36.43 ± 0.54b	87.12 ± 0.75c
Sitost	2.11 ± 0.10	2.39 ± 0.24b	2.05 ± 0.20ab	1.91 ± 0.10a	nd	nd	nd	1.36 ± 0.03a	1.00 ± 0.84a	3.00 ± 0.63b
Δ5-Avenast	1.66 ± 0.08	4.18 ± 0.25b	2.48 ± 1.00a	2.33 ± 0.70a	1.54 ± 0.38a	2.04 ± 0.15ab	2.44 ± 0.04b	1.16 ± 0.11b	0.82 ± 0.04a	2.44 ± 0.44c
Δ5,24-Stigmastadienol	2.07 ± 0.10	2.05 ± 0.56a	1.68 ± 0.30a	1.79 ± 0.50a	0.49 ± 0.11a	1.71 ± 0.07b	1.58 ± 0.02b	0.47 ± 0.01a	0.59 ± 0.05b	1.59 ± 0.07c
Δ7-Stigmastenol	0.62 ± 0.03	15.19 ± 0.71b	1.56 ± 0.90a	1.52 ± 0.90a	5.91 ± 0.05c	1.08 ± 0.06a	1.53 ± 0.07b	4.26 ± 0.16c	0.79 ± 0.06a	1.57 ± 0.05b
Δ7-Avenasterol	0.30 ± 0.02	3.09 ± 0.27b	0.40 ± 0.30a	0.36 ± 0.30a	2.04 ± 0.08b	3.69 ± 0.09c	0.45 ± 0.05a	1.68 ± 0.13b	0.31 ± 0.02a	0.39 ± 0.33a
Others	nd	nd	nd	nd	0.27 ± 0.04a	1.46 ± 0.05b	0.27 ± 0.03a	0.19 ± 0.01a	0.97 ± 0.84a	3.13 ± 0.63b
Total Sterols (mg/kg)	2515 ± 126	3963 ± 100b	2707 ± 111a	2703 ± 87a	3494 ± 46b	2282 ± 460a	2146 ± 174a	3509 ± 34c	3036 ± 76b	2498 ± 77a

Results are expressed as means ± SD (*n* = 3). Different letters in the same row within the same food frying operations (initial food, final food, and final OPO) indicate significant differences according to Tukey´s test at *p* < 0.05; nd: not detected.

**Table 9:** Bioactive compounds in initial and final olive-pomace oils and in initial and fried food lipids.

	OPO	French Fries			Croquettes			Nuggets		
	Initial	Initial	Fried	Final OPO	Initial	Fried	Final OPO	Initial	Fried	Final OPO
Tocopherols (mg/kg)	496 ± 13	194 ± 2b	102 ± 24a	93 ± 22a	nd	22 ± 30a	115 ± 27b	187 ± 10a	82 ± 10a	117 ± 81a
Squalene (mg/kg)	1062 ± 32	66 ± 3a	715 ± 21b	804 ± 18c	17 ± 2a	362 ± 15b	596 ± 21c	58 ± 5a	358 ± 62b	601 ± 44c
Triterpenic Alcohols (mg/kg)	708 ± 45	nd	638 ± 29a	664 ± 7a	nd	320 ± 11a	609 ± 33b	nd	281 ± 16a	532 ± 23b
Erythrodiol	636 ± 40	nd	574 ± 27a	596 ± 5a	nd	294 ± 20a	562 ± 23b	nd	259 ± 26a	574 ± 33b
Uvaol	72 ± 5	nd	65 ± 2a	68 ± 2a	nd	26 ± 4a	47 ± 5b	nd	22 ± 3a	58 ± 4b
Triterpenic Acids (mg/kg)	221 ± 11	33 ±2a	175 ± 8c	198 ± 3b	31 ± 0a	96 ± 12b	192 ± 11c	4 ± 1a	97 ± 6b	180 ± 6c
Oleanolic acid	201 ± 10	31 ± 2a	173 ± 8c	196 ± 3b	15 ± 0a	93 ± 12b	188 ± 11c	4 ± 1a	94 ± 6b	174 ± 11c
Ursolic acid	15 ± 1	2.0 ± 0.1a	2.0 ± 0.1a	2.0 ± 0.1a	15 ± 3c	2.0 ± 0.1a	4.0 ± 0.1b	nd	2.0 ± 0.1a	4.0 ± 0.1b
Maslinic acid	5.0 ± 0.2	nd	nd	nd	nd	1.0 ± 0.1	nd	nd	1.0 ± 0.1a	2.0 ± 0.1b
Aliphatic Alcohols (mg/kg)	1974 ± 56	nd	1945 ± 103a	1839 ± 211a	nd	1133 ± 63a	1906 ± 16b	nd	1103 ± 22a	1960 ± 49b

Results are expressed as means ± SD (*n* = 3). Different letters in the same row within the same food frying operations (initial food, final food, and final OPO) indicate significant differences according to Tukey´s test at *p* < 0.05; nd: not detected.

## Data Availability

Not applicable.

## Supplementary Materials

The following are available online at https://www.mdpi.com/article/10.3390/foods10122906/s1, Figure S1: GC chromatogram of sterols and triterpenic alcohols from olive pomace oil, Figure S2: GC chromatogram of aliphatic alcohols from olive pomace oil.
