# Supplementary material for: Stability of Bioactive Compounds in Olive-Pomace Oil at Frying Temperature and Incorporation into Fried Foods
Source: Foods. 2021 Nov 24;10(12):2906. doi: 10.3390/foods10122906 (PMC8700722; doi:10.3390/foods10122906)
Supplement: Supplementary file 1 [file foods-10-02906-s001.zip › foods-1462533-supplementary.pdf]

foods

MDPI

Figure S1. GC chromatogram of sterols and triterpenic alcohols from olive pomace oil

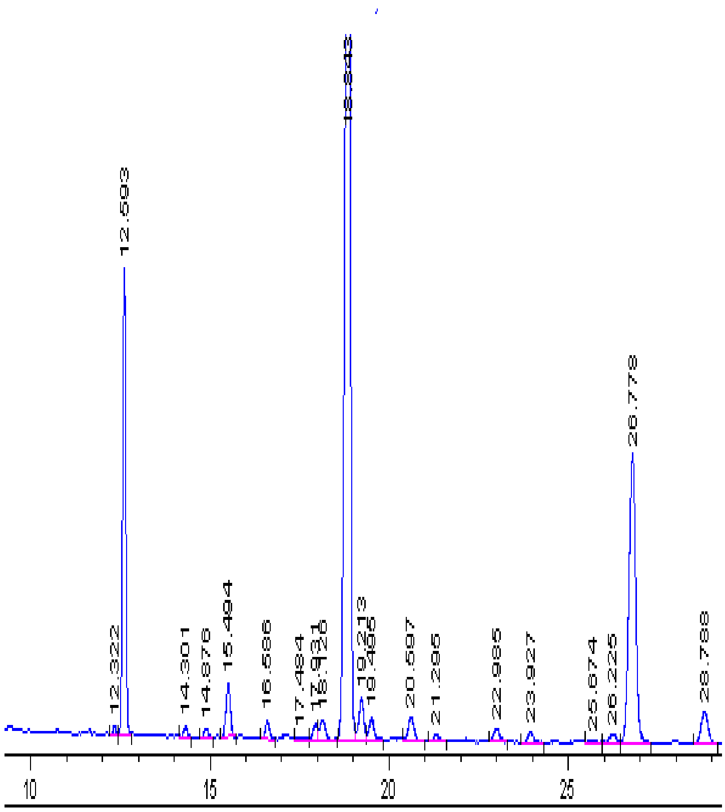

Retention time (min)	Assigned peak
**12,33**	Cholesterol
**12,59**	Cholestanol
**14,30**	Brasicasterol
**14,88**	24-Me-Cholesterol
**15,49**	Campesterol
**16,58**	Stigmasterol
**17,50**	Δ7-Campesterol
**17,93**	Δ5,23-Stigmastadienol
**18,60**	Clerosterol
**18,84**	β-Sitosterol
**19,21**	Sitostanol
**19,50**	Δ5-Avenasterol
**20,59**	Δ5,24-Stigmastadienol
**21,30**	Δ7-Stigmastenol
**22,11**	Δ7-Avenasterol
**26,70**	Erythrodiol
**28,79**	Uvaol

Capillary Column:	Bonded-phase 5% diphenyl-95% dimethylpolysiloxane;<br>30-m x 0.25-mm (i.d.) x 0.25-mm (film thickness)<br>(HP-5MS Agilent, prod. No. 19091 J-413)
Carrier Gas:	Hydrogen at a constant pressure, 15 psi.
Injector Temperature:	275°C
Detector Temperature:	320°C
Oven Temperature:	260°C

Figure S2. GC chromatogram of aliphatic alcohols from olive pomace oil

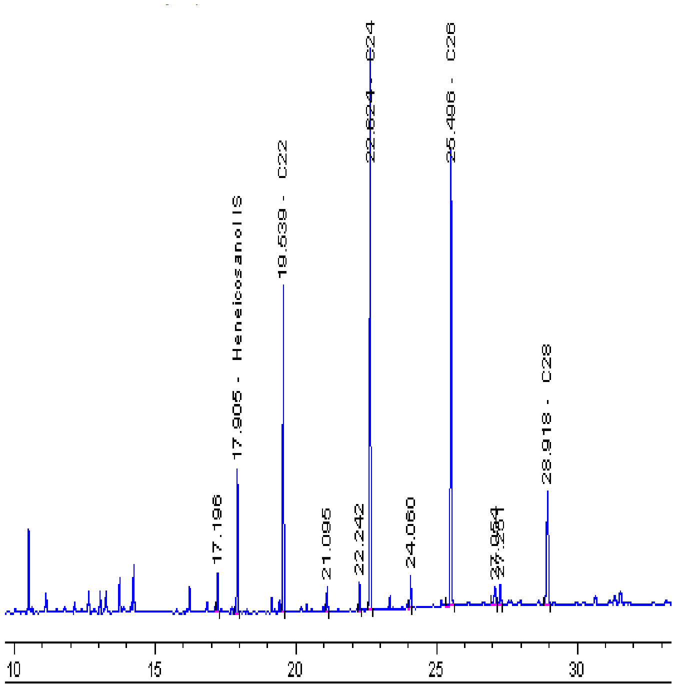

Retention time (min)	Assigned peak
**17.19**	Eicosanol
**17.90**	Heneicosanol (I.S.)
**19.53**	Docosanol
**21.09**	Tricosanol
**22.62**	Tetracosanol
**24.06**	Pentacosanol
**25.49**	Hexacosanol
**27.05**	Heptacosanol
**28.91**	Octacosanol

Capillary Column:	Bonded-phase 5% diphenyl-95%dimethylpolysiloxane;<br>30-m x 0.25-mm (i.d.) x 0.25-mm (film thickness)<br>(HP-5MS Agilent, prod. No. 19091 J-413)
Carrier Gas:	Hydrogen at a constant pressure, 15 psi.
Injector Temperature:	275°C
Detector Temperature:	300°C
Oven Temperature:	180°C
